# Moscatilin Inhibits Growth of Human Esophageal Cancer Xenograft and Sensitizes Cancer Cells to Radiotherapy

**DOI:** 10.3390/jcm8020187

**Published:** 2019-02-05

**Authors:** Wun-Ke Chen, Chien-An Chen, Chih-Wen Chi, Li-Hui Li, Chin-Ping Lin, Hui-Ru Shieh, Ming-Ling Hsu, Chun-Chuan Ko, Jeng-Jong Hwang, Yu-Jen Chen

**Affiliations:** 1 Department of Radiation Oncology, Hsinchu MacKay Memorial Hospital, Hsinchu 30071, Taiwan; gundem87@gmail.com; 2 Department of Biomedical Imaging and Radiological Sciences, National Yang-Ming University, Taipei 11221, Taiwan; 3 Department of Radiation Oncology in Zhongxing Branch, Taipei City Hospital, Taipei 10341, Taiwan; kenk102000@yahoo.com.tw; 4 Department of Medical Research, MacKay Memorial Hospital, New Taipei City 25160, Taiwan; cwchid48906003@gmail.com (C.-W.C.); lihua.530@yahoo.com.tw (L.-H.L.); cplin66@gmail.com (C.-P.L.); ru123@mmh.org.tw (H.-R.S.); sabinehsu@gmail.com (M.-L.H.); kochunchuan1113@gmail.com (C.-C.K.); 5 Department of Nursing, MacKay Medical College, New Taipei City 25245, Taiwan; 6 Department of Medical Research, China Medical University Hospital, Taichung 40402, Taiwan; 7 Department of Radiation Oncology, MacKay Memorial Hospital, Taipei 10449, Taiwan

**Keywords:** moscatilin, human esophageal cancer, polo-like kinase 1, radiosensitization

## Abstract

Esophageal cancer prognosis remains poor in current clinical practice. We previously reported that moscatilin can induce apoptosis and mitotic catastrophe in esophageal cancer cells, accompanied by upregulation of polo-like kinase 1 (Plk1) expression. We aimed to validate in vitro activity and Plk1 expression in vivo following moscatilin treatment and to examine the treatment’s radiosensitizing effect. Human esophageal cancer cells were implanted in nude mice. Moscatilin was intraperitoneally (i.p.) injected into the mice. Tumor size, body weight, white blood cell counts, and liver and renal function were measured. Aberrant mitosis and Plk1 expression were assessed. Colony formation was used to measure survival fraction after radiation. Moscatilin significantly suppressed tumor growth in mice bearing human esophageal xenografts without affecting body weight, white blood cell counts, or liver and renal function. Moscatilin also induced aberrant mitosis and apoptosis. Plk1 expression was markedly upregulated in vivo. Moreover, moscatilin pretreatment enhanced CE81T/VGH and BE3 cell radioresponse in vitro. Moscatilin may inhibit growth of human esophageal tumors and sensitize esophageal cancer cells to radiation therapy.

## 1. Introduction

Esophageal cancer is a health concern worldwide. Radiotherapy is one mainstream approach for treating esophageal cancer, as a neoadjuvant, adjuvant, or definitive therapy. Despite preoperative, perioperative, and postoperative efforts, the overall prognosis remains poor, with a 5-year overall survival of less than 40%. Median survival drops from 35 months for patients with local disease to 6 months for patients with metastatic disease [1,2,3]. A combination of chemotherapy with radiotherapy, termed chemoradiotherapy, has been reported to be superior to radiotherapy alone in treating patients with locally advanced esophageal cancer [4]. Clinical outcome remains poor, however, with a >40% failure rate and 50% mortality within 6 months [5]. It is, therefore, imperative to identify novel agents or effective sensitizers to overcome this problem, which may lead to better treatment strategies and further improve therapeutic outcomes.

Moscatilin (4,4’-dihydroxy-3,3’,5-trimethoxybibenzyl) is a bioactive compound derived from the Indian orchid *Dendrobium* and the stem of *Dendrobium loddigesii*. Previous studies have reported that this compound inhibits platelet aggregation [6], exerts anti-inflammatory [7] and antioxidant effects [8]. Recently, moscatilin was reported to exert potent cytotoxic effects and inhibit migration and angiogenesis in breast, lung, and colorectal cancer cells [9,10,11]. In preliminary work, we found that moscatilin suppressed the growth of esophageal cancer cells by inducing apoptosis and mitotic catastrophe concomitantly with increased expression of polo-like kinase 1 (Plk1) [12]. Mitotic catastrophe refers to either cell death or to a process preceding apoptosis, necrosis, or cell senescence to prevent the proliferation of defective mitotic cells [13,14]. Cell cycle-specific proteins such as polo-like kinases appear to be involved in mitotic catastrophe [15], Plk1 plays a critical role in cell division, and its expression and activity levels peak at mitosis [16].

In the present study, we investigated whether moscatilin would suppress esophageal cancer cell growth and increase Plk1 expression in vivo. As moscatilin has been shown to arrest esophageal cancer cells in the G_2_/M phase, which is sensitive to radiotherapy, we examined the effect of radiosensitization on cells in vitro.

## 2. Experimental Section

### 2.1. Esophageal Tumor Xenograft Animal Model

All experimental protocols involving animals were reviewed and approved by the Experimental Animal Committee of MacKay Memorial Hospital (approval number: MMH-A-S-102-68). All animal care and husbandry were conducted in accordance with the Mackay Memorial Hospital Guide for the Care and Use of Laboratory Animals (revised 2013). Male nude mice aged 4–5 weeks were obtained from the National Laboratory Animal Center (Taipei, Taiwan) and housed in a rodent facility at 22 ± 1 °C with a 12 h light/dark cycle. After arrival, the animals were kept in our animal facilities for acclimatization for 7 days, with free access to food and water. Approximately 10^6^ human esophageal CE81T/VGH cancer cells in 0.1 mL of phosphate-buffered saline (PBS) were subcutaneously injected into the right back gluteal region. The tumors were allowed to grow for 21 days to approximately 0.5 cm in diameter.

### 2.2. In Vivo Therapeutic Study

Animals were randomized into two groups: Untreated controls and moscatilin treatment. Moscatilin was kindly provided by Prof. Chen (Department of Biotechnology, Hung Kuang University, Taichung, Taiwan) that was extracted and purified from *Dendrobium loddigesii.* The purity of moscatilin was examined by high-performance liquid chromatography and nuclear magnetic resonance and is more than 98% [6]. Moscatilin was dissolved in dimethyl sulfoxide (DMSO) as stock concentration of 50 mg/mL. Moscatilin at a dose of 50 mg/kg was intraperitoneally (i.p.) injected into the mice with the volume of 25 μL. Control mice received equal amounts of vehicle. All implanted tumors were measured by one observer. Calipers were used to measure the largest (a) and smallest (b) diameters, and the tumor volumes were estimated according to the formula 0.5 × a × b^2^ [17].

### 2.3. Toxicity Assessment

Mice were examined twice a week for changes in body weight and immunological and hematological indicators. Immunological parameters were white blood cell (WBC) counts measured using the retro-orbital blood sampling method in a Hemavet blood analyzer (Drew Scientific, Oxford, CT, USA). Hematological toxicity was assessed as alanine aminotransferase (ALT) and creatinine (CRE) levels, representing liver and kidney function, respectively. ALT and CRE levels were measured using a Fuji Dri-Chem 3500 analyzer (Fujifilm Medical Systems, Tokyo, Japan).

### 2.4. Histological and Immunohistochemistry (IHC) Analyses

After moscatilin treatment for 2 months, the survival rate of mice was 60% (two of five mice were died in each group during experiment). All survival mice were anesthetized with Zoletil 50 (25 mg/kg) plus Xylazine (10 mg/kg) and tumors were harvested immediately. The mice were then euthanized with carbon dioxide. Tumors were sliced into 4 μm-thick sections, fixed with 4% formalin, and stained with hematoxylin and eosin (H&E) according to standard procedures. For immunohistochemistry (IHC) analysis, tissues were fixed in formalin and embedded in paraffin, then cut into 4 µm-thick sections. Following deparaffinization, rehydration, and inactivation of endogenous peroxidase by hydrogen peroxide, slides were placed in Tris/EDTA buffer pH 9 with heat mediation. After washing with PBS and eliminating non-specific binding of the antibody with 2% normal serum, the sections were incubated with primary rabbit polyclonal Plk1 (phospho-S137) antibody (Abcam, Cambridge, UK) at 4 °C overnight. Next, slides were washed with phosphate-buffered saline (PBS) three times, and incubated with undiluted horseradish peroxidase (HRP)-conjugated anti-rabbit IgG as a secondary antibody. Antigen-antibody complexes were detected by the avidin-biotin-peroxidase method. Finally, sections were counterstained with hematoxylin and photographed using a light microscope at a magnification of 100× or 400×. PBS was used as the negative control.

### 2.5. Moscatilin Treatment and Radiation Delivery

Cells were plated onto culture dishes and grown in DMEM containing 10% fetal calf serum (FCS). After 24 h, the cells were treated with various concentrations of moscatilin 4 h prior to irradiation. Cells were then exposed to a specific dose of radiation. Radiation therapy with a 6-MeV electron beam energy was delivered by a linear accelerator (Clinac 1800, Varian Medical Systems, Palo Alto, CA, USA) at a rate of 2.4 Gy/min for various doses (0, 0.5, 1, 2, 4, and 6 Gy) in a single fraction. These radiation doses were selected following our preliminary work on calibration of radiation survival curves for CE81T/VGH and BE3 cells, to ensure adequate coverage from 100% to less than 37% survival (D0 in radiobiology) for further estimation of the surviving fraction. For clinical relevance, 2 Gy were also selected to match the daily fraction size commonly used in clinical practice. Full electron equilibrium was ensured for each fraction by a parallel plate PR-60C ionization chamber (CAPINTEL, Inc., Ramsey, NJ, USA).

### 2.6. Colony Forming Assay and Estimation of Sensitizer Enhancement Ratio (SER)

After radiation, cells were plated at a density of 10^3^ cells onto 6-well plates and allowed to grow in DMEM containing 10% FCS. After 10–14 days of incubation, the cells were stained with 3% crystal violet, and survival clones containing more than 50 cells were counted. The mean control plating efficiency for untreated CE81T/VGH squamous cell carcinoma (SCC) cells was approximately 37%. The surviving fraction was calculated as mean colonies divided by cells inoculated. A linear-quadratic model was used to fit survival curves. The sensitizer enhancement ratio (SER) was calculated as the radiation dose needed for radiation alone divided by the dose needed for various concentrations of moscatilin plus radiation at a surviving fraction of 37% (D0 in radiobiology).

### 2.7. Statistical Analysis

Results are expressed as mean ± standard error of mean (SEM). Statistical comparison in each experiment was performed using Student’s *t*-test or one-way analysis of variance (ANOVA). The difference was considered significant at *p* < 0.05. We used SigmaPlot version 8.0 (IBM SPSS, Armonk, NY, USA) with written syntax to fit survival curves using a linear quadratic model.

## 3. Results

### 3.1. Growth Inhibitory Effect on Tumor Xenografts

**Figure 1:**
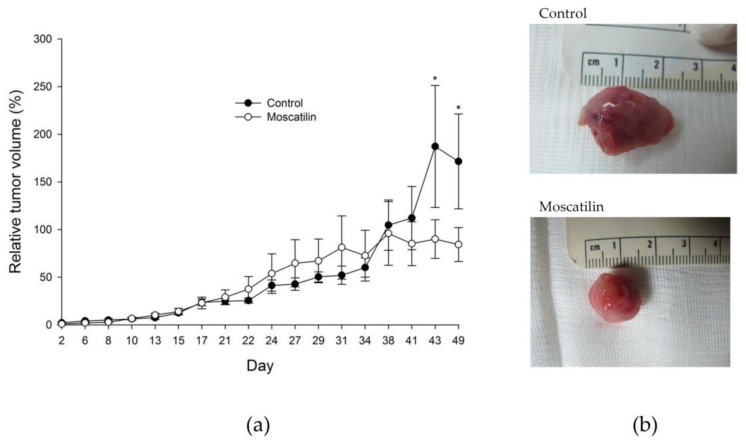
Moscatilin inhibited esophageal cancer tumor growth in vivo. The tumor size observed in the moscatilin-treated group (50 mg/kg) decreased significantly compared with tumor size in the control group * *p* < 0.05 (**a**). Representative photographs of tumor specimens of control and moscatilin-treated mice, as indicated (**b**).

**Figure 2:**
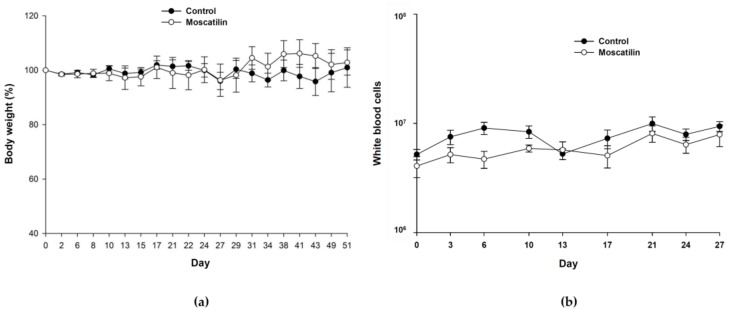
There were no significant differences in body weight growth kinetics between the control and moscatilin-treated groups (**a**). No marked effect was observed in white blood cell counts between the groups (**b**).

To validate the in vitro effects of moscatilin against esophageal cancer, CE81T/VGH cancer cells were xenografted into nude mice. The tumor-bearing nude mice were treated with either DMSO as the control or moscatilin (five mice per group). Plotted curves showed that tumor growth could be moderately retarded by moscatilin though not statistically significant (Figure 1a). Representative photographs of control and moscatilin-treated tumor specimens were indicated in Figure 1b. There were no significant adverse effects on body weight (Figure 2a) or WBC counts (Figure 2b). The nadir in WBC count of moscatilin-treated group is significantly lower than control group. There is no significant difference of ALT (Table 1) and CRE (Table 2) amounts between control and moscatilin-treated groups. ALT and CRE values fell within the normal range 28–132 U/L and 0.2–0.8 mg/dL, respectively.

### 3.2. Histology and IHC

**Figure 3:**
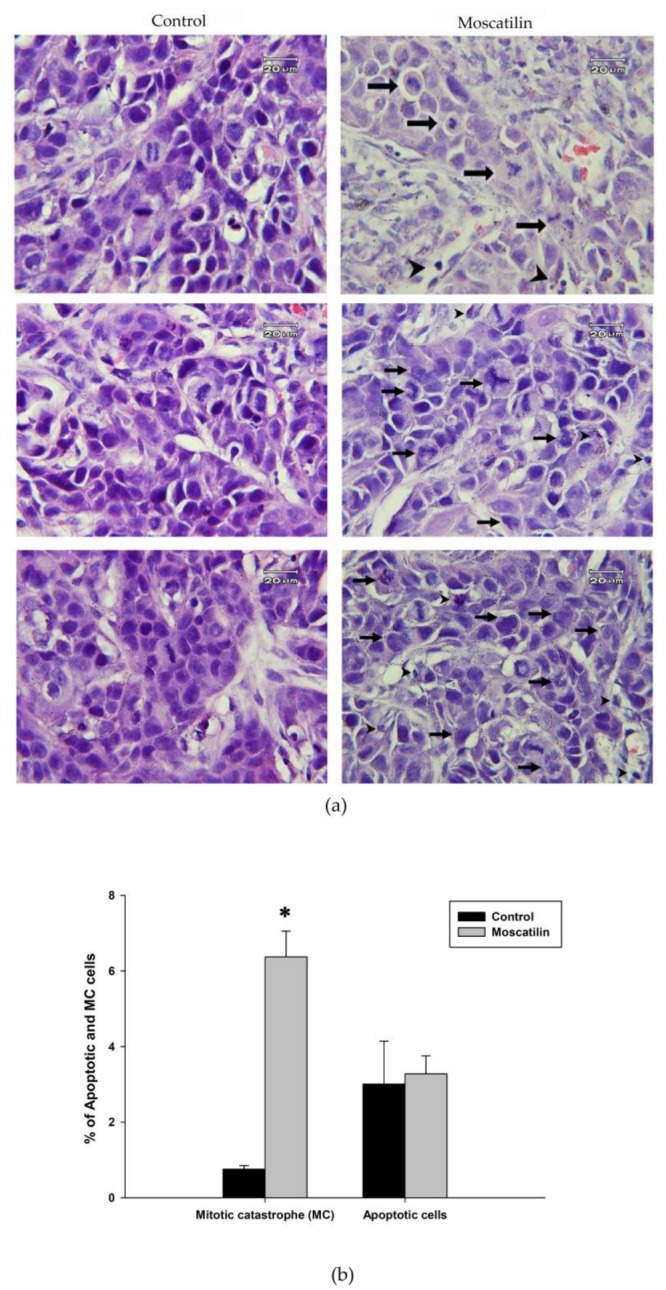
H&E-stained control and moscatilin-treatment for 60 days of (**a**) tumor samples. Aberrant mitotic cells with mitotic catastrophe (right panel arrows) and apoptotic cells (right panel arrowheads) were observed in tumor sections from moscatilin-treated mice but not in control mice. The quantification of mitotic catastrophe (MC) and apoptotic cells in control and moscatilin-treated tumor were shown in (**b**). Significant difference between control cells and cells treated with moscatilin are indicated by * *p* < 0.05.

**Figure 4:**
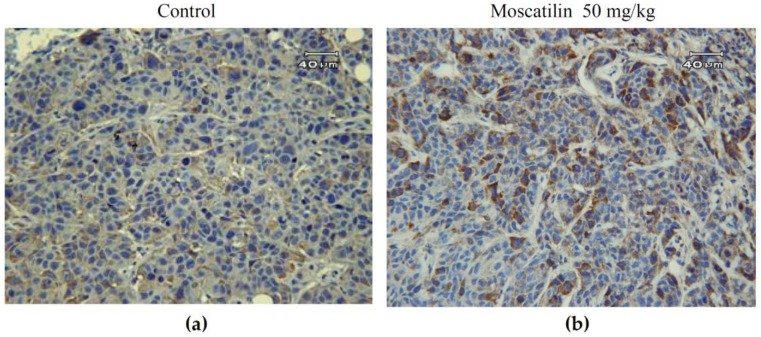
Representative immunohistochemistry slides showing phospho-Plk 1 protein expression in esophageal cancer CE81T/VGH xenograft tumor sections after treatment with control (dimethyl sulfoxide, DMSO) (**a**) or moscatilin (**b**) for 60 days.

Aberrant mitotic cells with spindle perturbation and mitotic catastrophe were observed in the H&E-stained slides of tumor samples from moscatilin-treated mice for 60 days but not in the slides from control mice (Figure 3a). The amounts of mitotic catastrophe increased 8.4-fold in moscatilin-treated group compared with control group. Slides from the moscatilin-treated group showed apoptotic cells with a round or oval condensed eosinophilic cytoplasm and dense nucleus chromatin fragments. Phospho-Plk1 expression in CE81T/VGH xenografts was demonstrated immunohistochemically staining by positive distribution on both nuclear and cytoplasm (Figure 4). Representative samples displayed markedly higher expression of phospho-Plk1 in the moscatilin-treated specimens than in the control group.

### 3.3. Radiosensitizing Activity In Vitro

**Figure 5:**
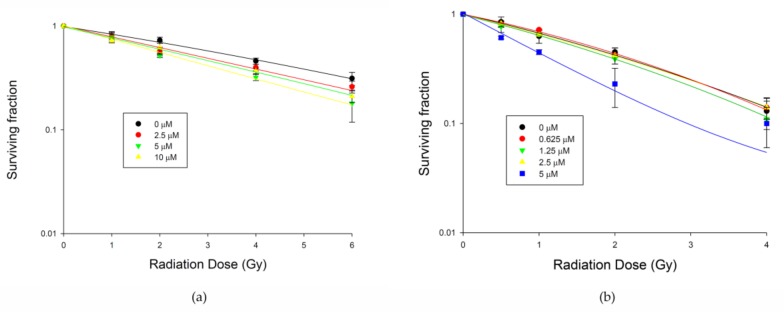
Moscatilin enhanced the radiosensitivity of human esophageal cancer cells. (**a**) BE3 cells. (**b**) CE81T/VGH cells. Clonogenic assay was used to estimate the survival of esophageal cancer cells. Significant difference between control cells and cells treated with moscatilin 10 μM in BE3 cells or 5 μM in CE81T/VGH cells from colony formation data using Student’s *t*-test analysis are indicated as * *p* < 0.05.

Clonogenic assays were performed to determine if moscatilin treatment exerted effects on radiation-induced tumor cell killing. Preincubation with 5 μM of moscatilin-sensitized BE3 cells to 6 Gy of radiation significantly, with a sensitizer enhancement ratio of 1.5 (Figure 5a), and significantly enhanced the effects of 2 Gy of radiation on CE81T/VGH cells, with a sensitizer enhancement ratio of 1.8 (Figure 5b). When compared to the original data from the colony formation assay, the significant differences between control cells and cells treated with moscatilin 10 μM in BE3 cells or 5 μM in CE81T/VGH cells are indicated as * *p* < 0.05.

## 4. Discussion

In the present study, moscatilin effectively suppressed tumor growth in vivo without exerting significant toxicity, and enhanced the sensitivity of BE3 and CE81T/VGH esophageal cancer cells to radiation in vitro, suggesting a potential new application of this compound in cancer therapy. Additional work is required to explore the clinical benefits and fully understand the mechanisms of moscatilin-mediated radiosensitization.

The platinum analog cisplatin is one of the most widely used and highly effective drugs for the treatment of esophageal cancer [18,19]. However, nephrotoxicity is a well-known and dose-limiting side effect that occurs in 20–30% of patients [20,21]. Presuming that a novel compound isolated from natural products would be relatively safe compared to a synthetic one, we designed in vivo xenograft experiments. Our results indicate that moscatilin may exert inhibitory effects against human esophageal carcinoma growth without overt toxicity in renal and liver function. Previously, we reported that cisplatin is effective on inhibiting esophageal tumor growth with moderate toxicity on hematological profile [22]. The potential of moscatilin combined with cisplatin as chemo-sensitizer to enhance cisplatin effect in esophageal cancer or combination with immunotherapy will be further tested in the future.

Despite recent advances, the effect of radiotherapy is limited by both tumor cell radioresistance and adverse reactions in the normal tissues surrounding the tumor. One strategy to improve the efficacy of radiotherapy is radiosensitizing the tumor cells without damaging normal tissue cells. A differential outcome of irradiation is effects on the cell cycle. In both the G_2_ and M phases, cells are more sensitive to radiation; mitotic cells have been shown to be the most sensitive [23,24,25]. In our previous study, moscatilin increased the percentage of phospho-histone H3-positive cells; we concluded that moscatilin induced mitotic arrest. Because Plk1 expression and activity increase during mitosis [16,26], we first checked Plk1 expression levels. Following moscatilin treatment, higher expression of phospho-Plk1 in CE81T/VGH xenografts was detected, consistent with our previous findings in vitro. This mitosis-arresting activity may be related to the major effects of moscatilin.

Mitotic arrest stemming from perturbation of the mitotic apparatus results in mitotic catastrophe [13,27]. Our previous study found that moscatilin evoked mitotic catastrophe in esophageal cancer cells. In the present study, tumor samples from moscatilin-treated mice displayed aberrant mitosis and some apoptotic cells. Moscatilin appears to be a potential oncosuppressive drug for the treatment of esophageal cancer.

## 5. Conclusions

In conclusion, the present study demonstrates that moscatilin can act as a novel anti-cancer agent when administered either alone or in combination with radiotherapy. Its potential application in cancer therapy merits further investigation.

## Figures and Tables

**Table 1:** Effects of treatment with control or moscatilin on alanine aminotransferase (ALT) and creatinine levels. Data are presented as mean ± SEM.

ALT (U/L)	Day 0	Day 3	Day 14	Day 28
Control	4.0 ± 3.10	20.4 ± 3.07	8.67 ± 8.01	3.33 ± 1.25
Moscatilin	5.8 ± 5.38	25.2 ± 15.12	11.33 ± 6.6	6.67 ± 3.09

ALT: alanine aminotransferase.

**Table 2:** Effects of treatment with control or moscatilin on creatinine levels. Data are presented as mean ± SEM.

CRE (mg/dL)	Day 0	Day 3	Day 14	Day 28
Control	0.28 ± 0.19	0.34 ± 0.08	0.47 ± 0.12	0.6 ± 0.22
Moscatilin	0.54 ± 0.05	0.4 ± 0.06	0.6 ± 0.22	0.43 ± 0.0

Cre: creatinine.
